# Supplementary material for: Coordinated and Distinct Roles of Peptidoglycan Carboxypeptidases DacC and DacA in Cell Growth and Shape Maintenance under Stress Conditions
Source: Microbiol Spectr. 2023 Apr 26;11(3):e00014-23. doi: 10.1128/spectrum.00014-23 (PMC10269652; doi:10.1128/spectrum.00014-23)
Supplement: Supplemental file 1 — Fig. S1 to S9 and Tables S1 and S2. Download spectrum.00014-23-s0001.pdf, PDF file, 1.6 MB [file spectrum.00014-23-s0001.pdf]

Supplementary materials

1. Supplementary Figures

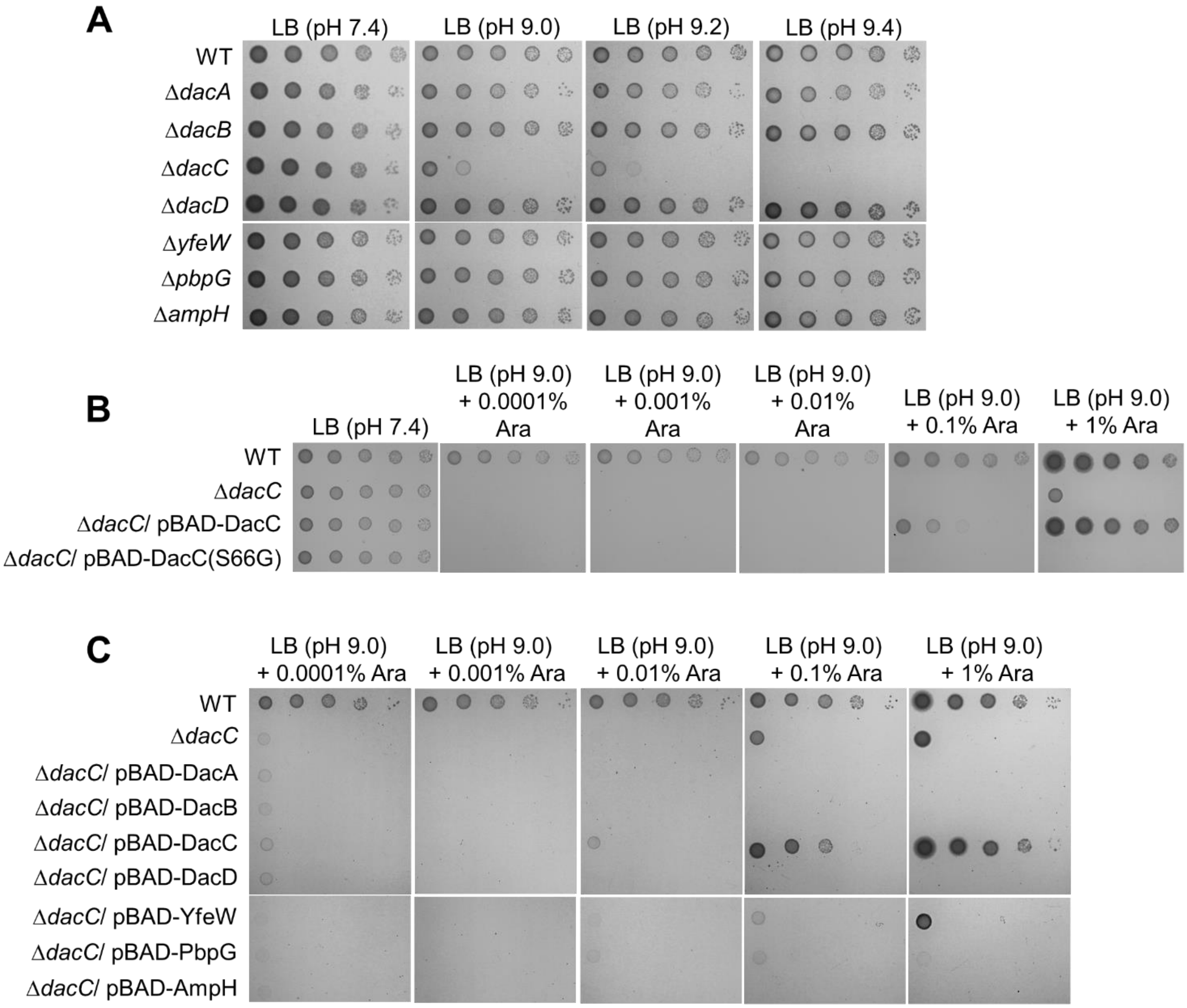

FIG S1 The activity of DacC is required for growth under alkaline stress conditions. (A) Sensitivity of the *dacC* mutant to alkaline pH. The wild-type (WT) and indicated mutant cells were serially diluted from 10^8 to 10^4 cells/ml in 10-fold steps and spotted onto an LB plate or LB plates at indicated pH. (B) Complementation of alkaline sensitivity of the *dacC* mutant. The cells of the indicated strains were serially diluted from 10^8 to 10^4 cells/ml in 10-fold steps and spotted onto an LB plate and LB plates at pH 9.0 containing the indicated concentrations of arabinose. (C) Complementation of alkaline sensitivity of the *dacC* mutant by other DD-CPases. The cells of the indicated strains were serially

diluted from 10^8 to 10^4 cells/ml in 10-fold steps and spotted onto an LB plate and LB plates at pH 9.0 containing the indicated concentrations of arabinose.

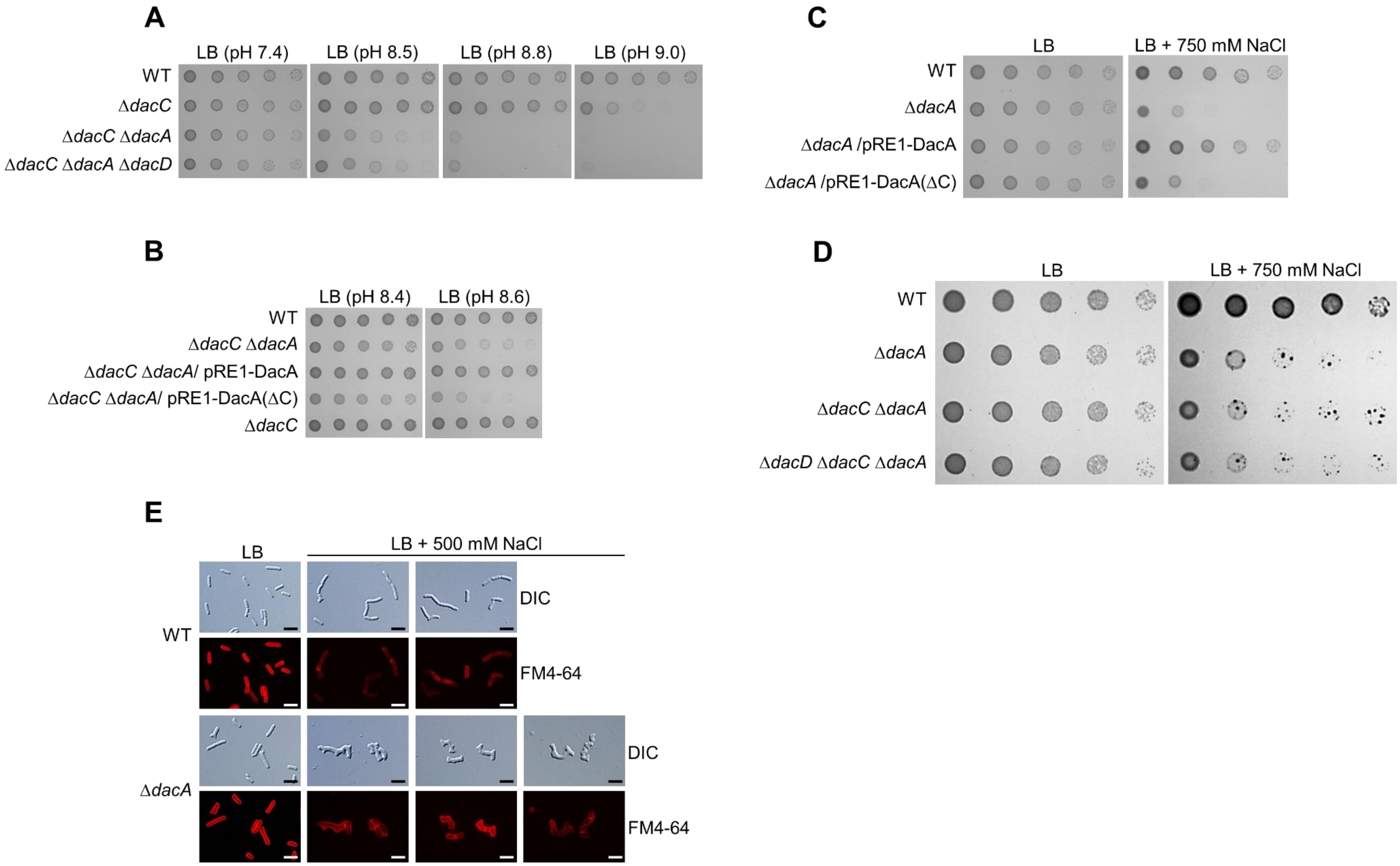

FIG S2 Effects of DacA depletion on cell growth and morphology under alkaline and salt stresses. (A) Increased sensitivity of the *dacC* mutant to alkaline pH by additional deletion of the *dacA* gene. The wild-type (WT) and indicated mutant cells were serially diluted from 10^8 to 10^4

cells/ml in 10-fold steps and spotted onto an LB plate or LB plates at indicated pH. (B) Complementation of alkaline sensitivity of the *dacC dacA* double mutant. The cells of the indicated strains were serially diluted from 10^8 to 10^4 cells/ml in 10-fold steps and spotted onto an LB plate and LB plates at the indicated pH. (C) Salt sensitivity of the *dacA* mutant. The wild-type and indicated mutant cells were serially diluted from 10^8 to 10^4 cells/ml in 10-fold steps and spotted onto an LB plate (171 mM NaCl) or an LB plate containing 750 mM NaCl. (D) Only DacA is required for overcoming salt stress. The wild-type and indicated mutant cells were serially diluted from 10^8 to 10^4 cells/ml in 10-fold steps and spotted onto an LB plate or an LB plate containing 750 mM NaCl. (E) Effect of DacA depletion on morphological maintenance under salt stress. The indicated cells grown in LB medium or LB medium containing 750 mM NaCl were stained with FM4-64 (red), and then spotted on a 1% agarose pad. Scale bars, 5 μm.

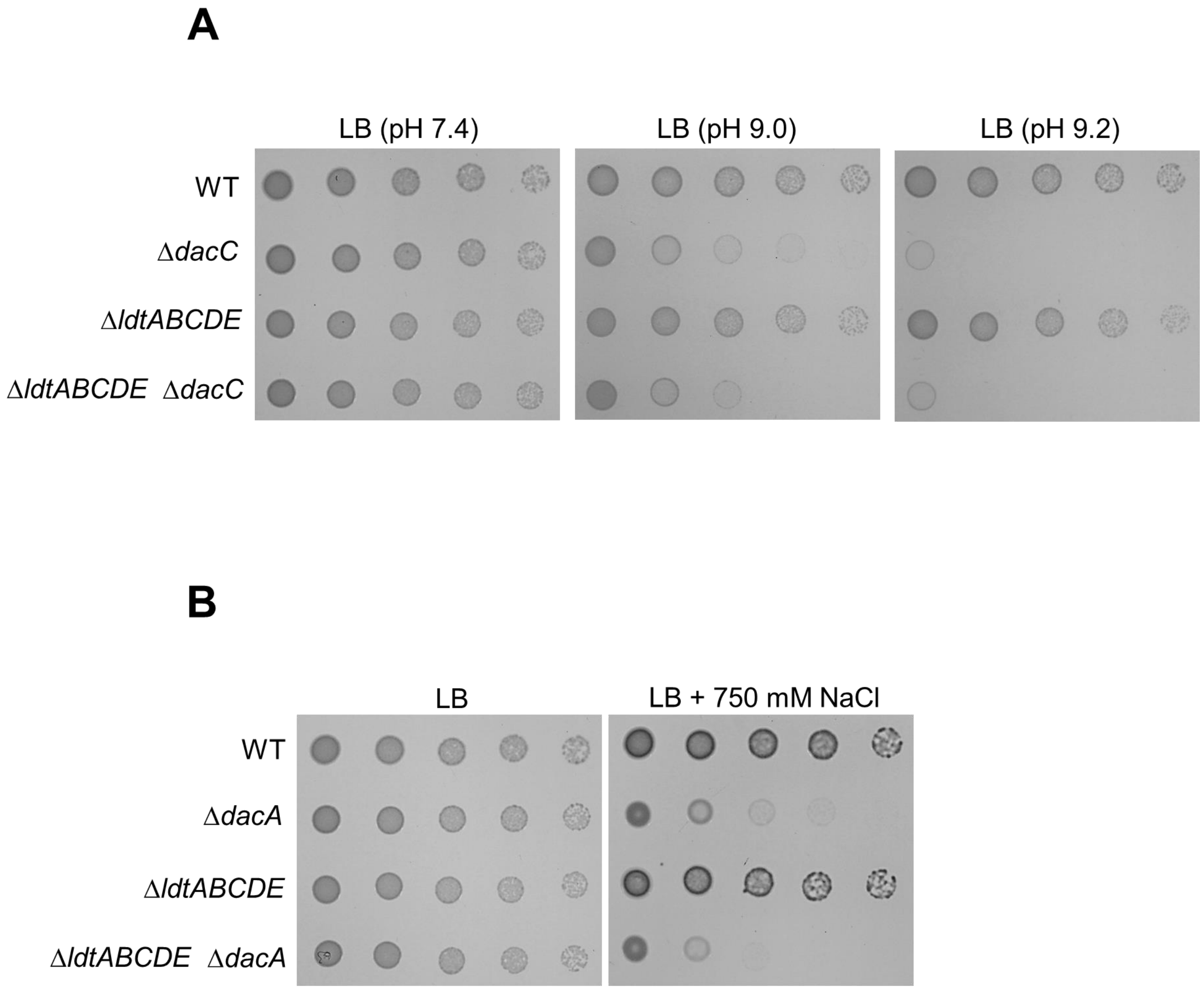

FIG S3 Identified phenotypes of the *dacC* and *dacA* mutants are independent of LD-transpeptidases. (A) LD-Transpeptidase-independent alkaline sensitivity of the *dacC* mutant. The wild-type (WT) and indicated mutant cells were serially diluted from 10^8 to 10^4 cells/ml in 10-fold steps and spotted onto an LB plate or LB plates at indicated pH. (B) LD-Transpeptidase-independent salt sensitivity of the *dacA* mutant. The wild-type and indicated mutant cells were serially diluted from 10^8 to 10^4 cells/ml in 10-fold steps and spotted onto an LB plate or an LB plate containing 750 mM NaCl.

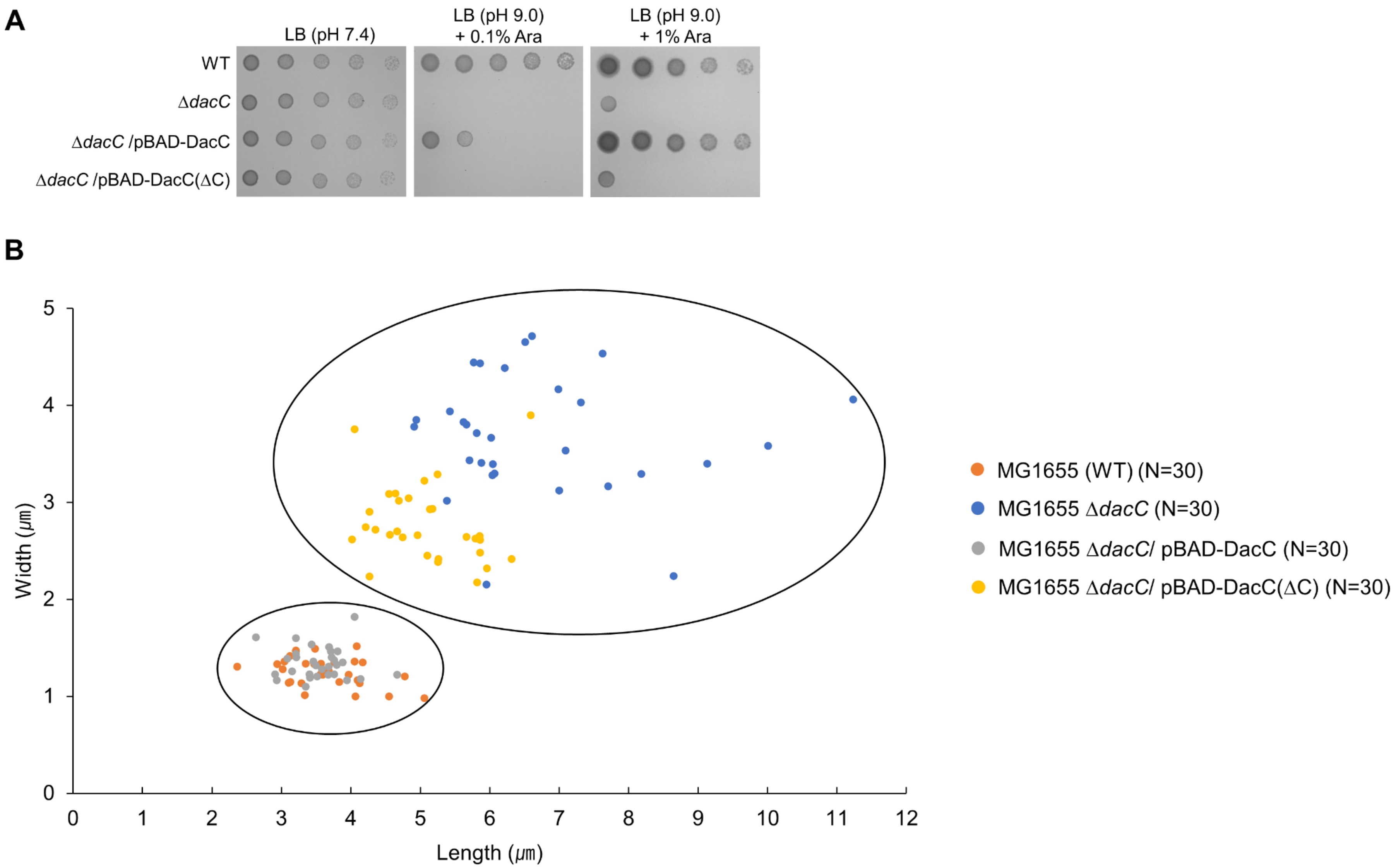

FIG S4 The C-terminal domain of DacC is necessary for cell growth and morphology under alkaline stress. (A) The C-terminal domain of DacC is necessary for bacterial growth under alkaline stress. The wild-type (WT) and indicated mutant cells were serially diluted from 10^8 to

10^4 cells/ml in 10-fold steps and spotted onto an LB plate or LB plate at pH 9.0 containing 0.1% or 1% arabinose. (B) Length and width distribution for cells grown in LB medium at pH 9.0. N indicates the number of cells.

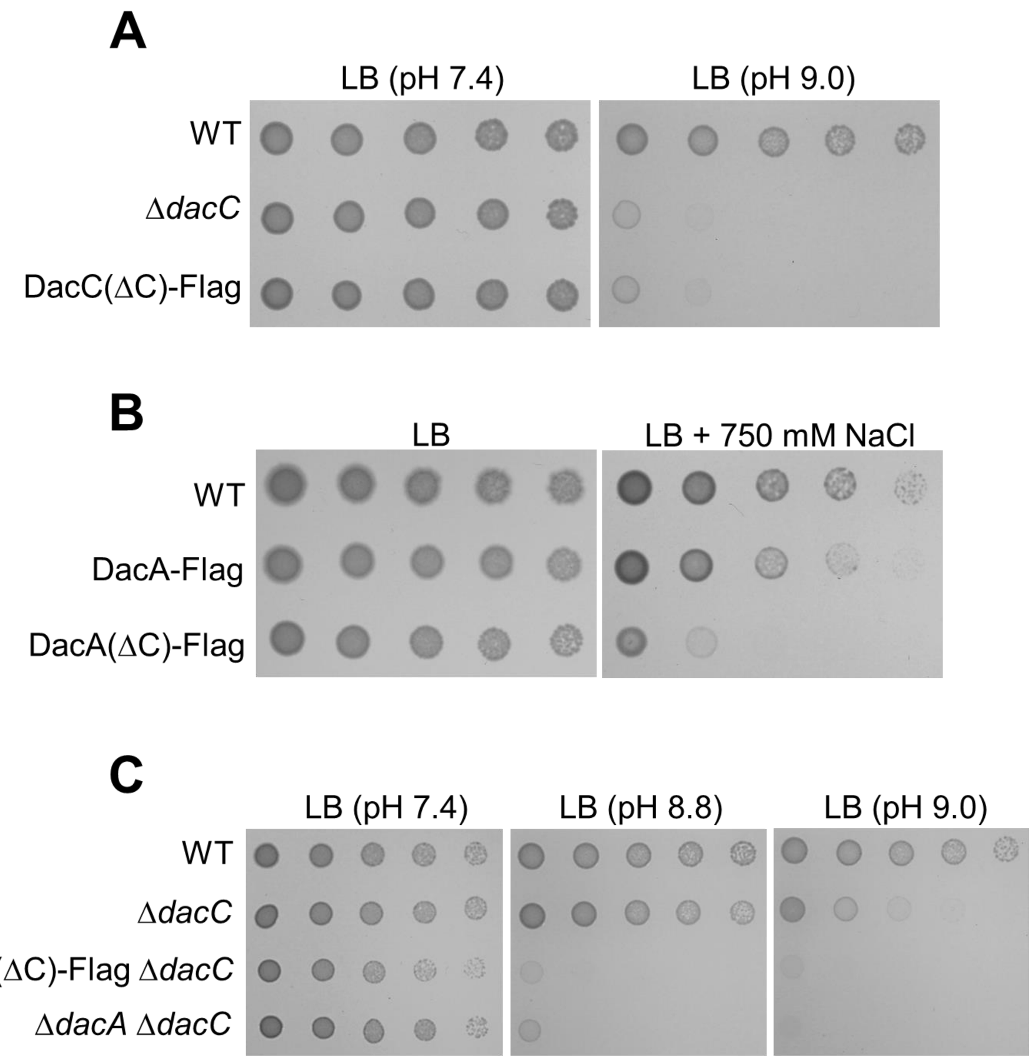

FIG S5 Phenotypes of the MG1655 DacC(ΔC)-Flag and MG1655 DacA(ΔC)-Flag strains. (A) Sensitivity of the MG1655 DacC(ΔC)-Flag strain to alkaline pH. The wild-type (WT) and indicated mutant cells were serially diluted from 10^8 to 10^4 cells/ml in 10-fold steps and spotted onto an LB plate or LB plate at pH 9.0. (B) Salt sensitivity of the MG1655 DacA(ΔC)-Flag strain. The wild-type and indicated mutant cells were serially diluted from 10^8 to 10^4 cells/ml in 10-fold steps and spotted onto an LB plate or LB plate containing 750 mM NaCl. (C) Phenotype of the MG1655 DacA(ΔC)-Flag Δ*dacC* strain under alkaline stress conditions. The wild-type and indicated mutant cells were serially diluted from 10^8 to 10^4 cells/ml in 10-fold steps and spotted onto an LB plate or LB plates at the indicated pH.

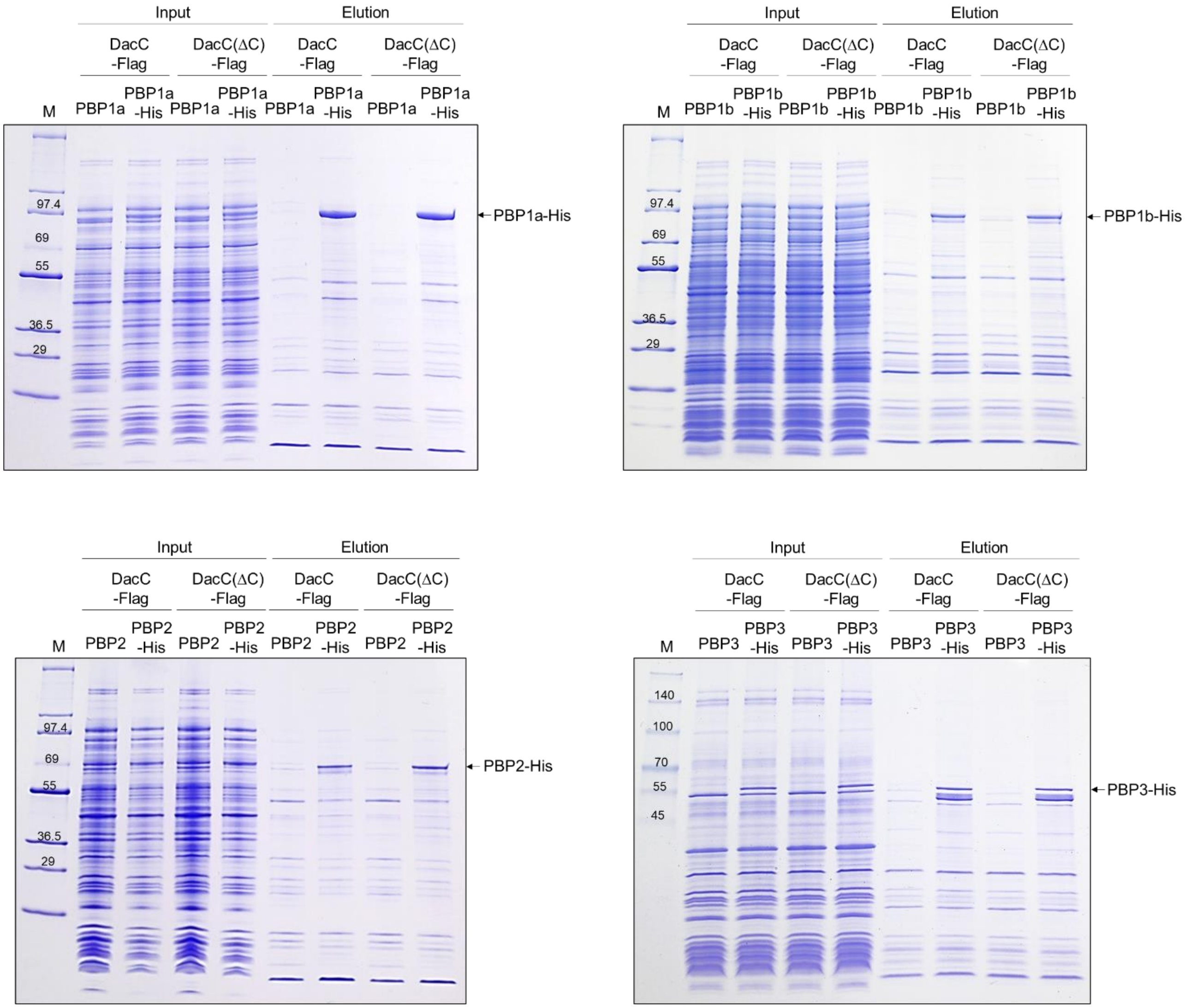

FIG S6 SDS-PAGE gels of pull-down experiments of DacC. The supernatant of MG1655 DacC-Flag or DacC(ΔC)-Flag cells harboring the pBAD-based plasmids expressing C-terminal His-tagged PBPs or non-tagged PBPs was loaded onto Talon metal affinity resin. After pull-down experiments, the amount of input (Input) and output (Elution) total proteins was measured by SDS-PAGE using 4–20% gradient Tris-glycine polyacrylamide gels (KOMA biotech, Korea). Arrows indicate purified His-tagged PBPs. Lane M indicates EzWay Protein Blue MW Marker (KOMA Biotech., Korea) or EzWay Protein PreBlue Ladder (KOMA Biotech., Korea).

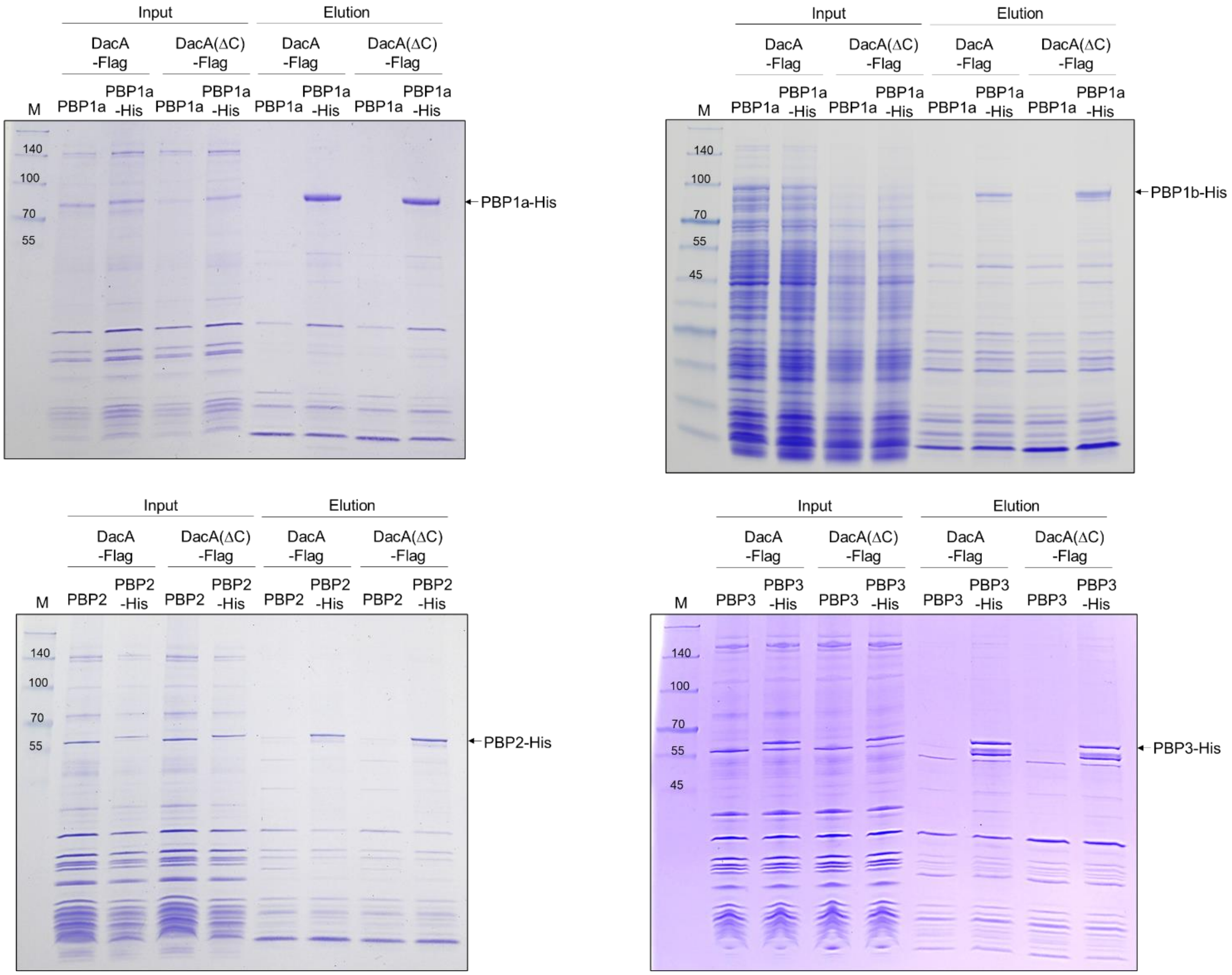

FIG S7 SDS-PAGE gels of pull-down experiments of DacA. The supernatant of MG1655 DacA-Flag or DacA(ΔC)-Flag cells harboring the pBAD-based plasmids expressing C-terminal His-tagged PBPs or non-tagged PBPs was loaded onto Talon metal affinity resin. After pull-down experiments, the amount of input (Input) and output (Elution) total proteins was measured by SDS-PAGE using 4–20% gradient Tris-glycine polyacrylamide gels (KOMA biotech, Korea). Arrows indicate purified His-tagged PBPs. Lane M indicates EzWay Protein PreBlue Ladder (KOMA Biotech., Korea).

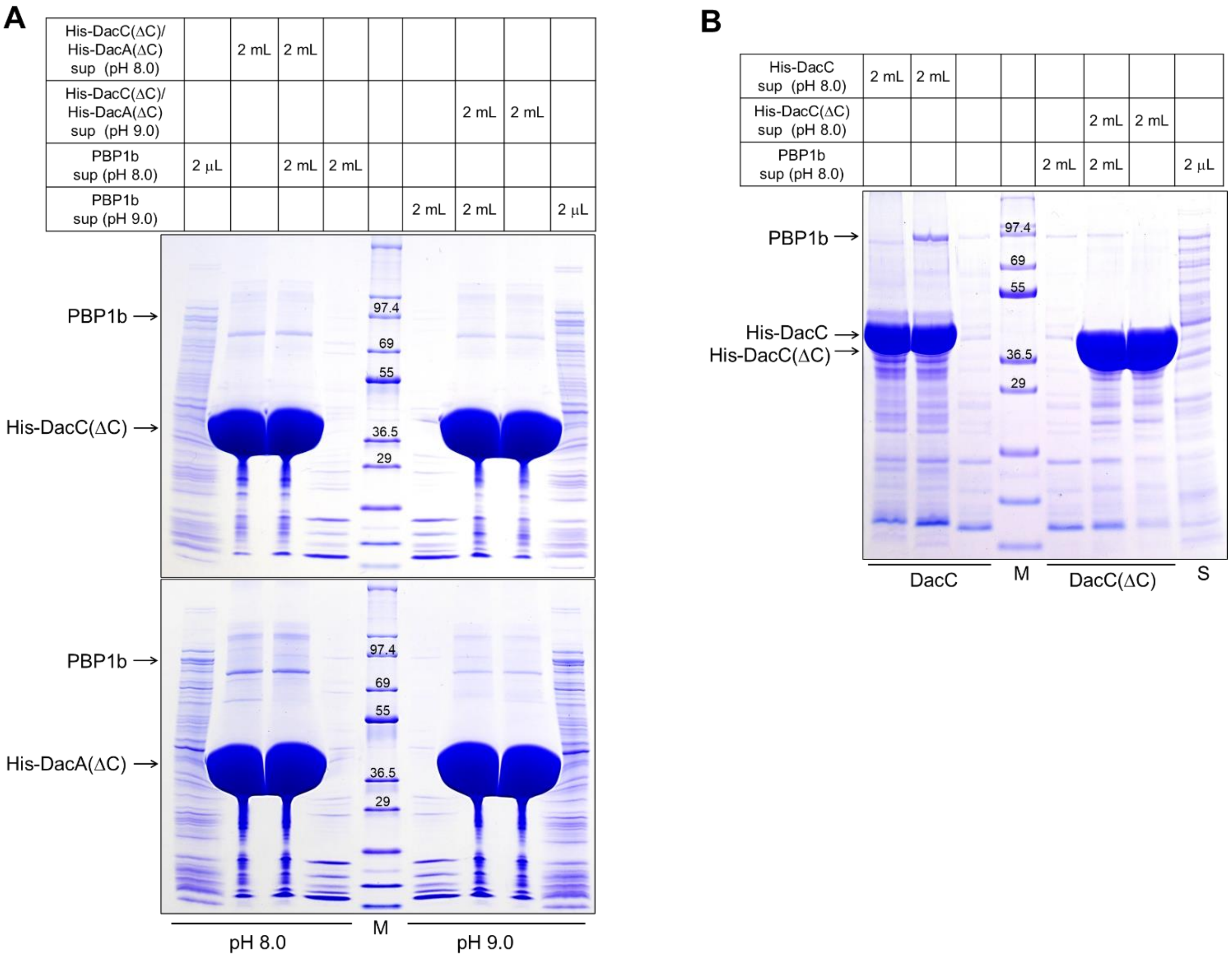

FIG S8 The importance of the C-terminal domain of DacC and DacA for its interaction with PBP1b. (A) PBP1b does not interact with DacC(ΔC) and DacA(ΔC) regardless of pH conditions. The supernatant of ER2566 cells harboring the pET24a plasmid expressing PBP1b was mixed with the supernatant of ER2566 cells harboring pET28a plasmid expressing His-tagged DacC(ΔC) or His-tagged DacA(ΔC) at pH 8.0 or 9.0. After pull-down experiments, eluted proteins were separated on 4–20% gradient Tris-glycine polyacrylamide gels and were visualized by staining with Coomassie brilliant blue R. Lane M indicates EzWay Protein Blue MW Marker (KOMA Biotech., Korea). Lane S indicates the supernatant of ER2566 cells overexpressing PBP1b. (B) PBP1b interacts with DacC, but not DacC(ΔC). The supernatant of ER2566 cells harboring the pET24a plasmid expressing PBP1b was mixed with the supernatant of ER2566 cells harboring pET28a plasmid expressing His-tagged

DacA or His-tagged DacA(ΔC) at pH 8.0. After pull-down experiments, eluted proteins were separated on 4–20% gradient Tris-glycine polyacrylamide gels and were visualized by staining with Coomassie brilliant blue R. Lane M indicates EzWay Protein Blue MW Marker (KOMA Biotech., Korea). Lane S indicates the supernatant of ER2566 cells overexpressing PBP1b.

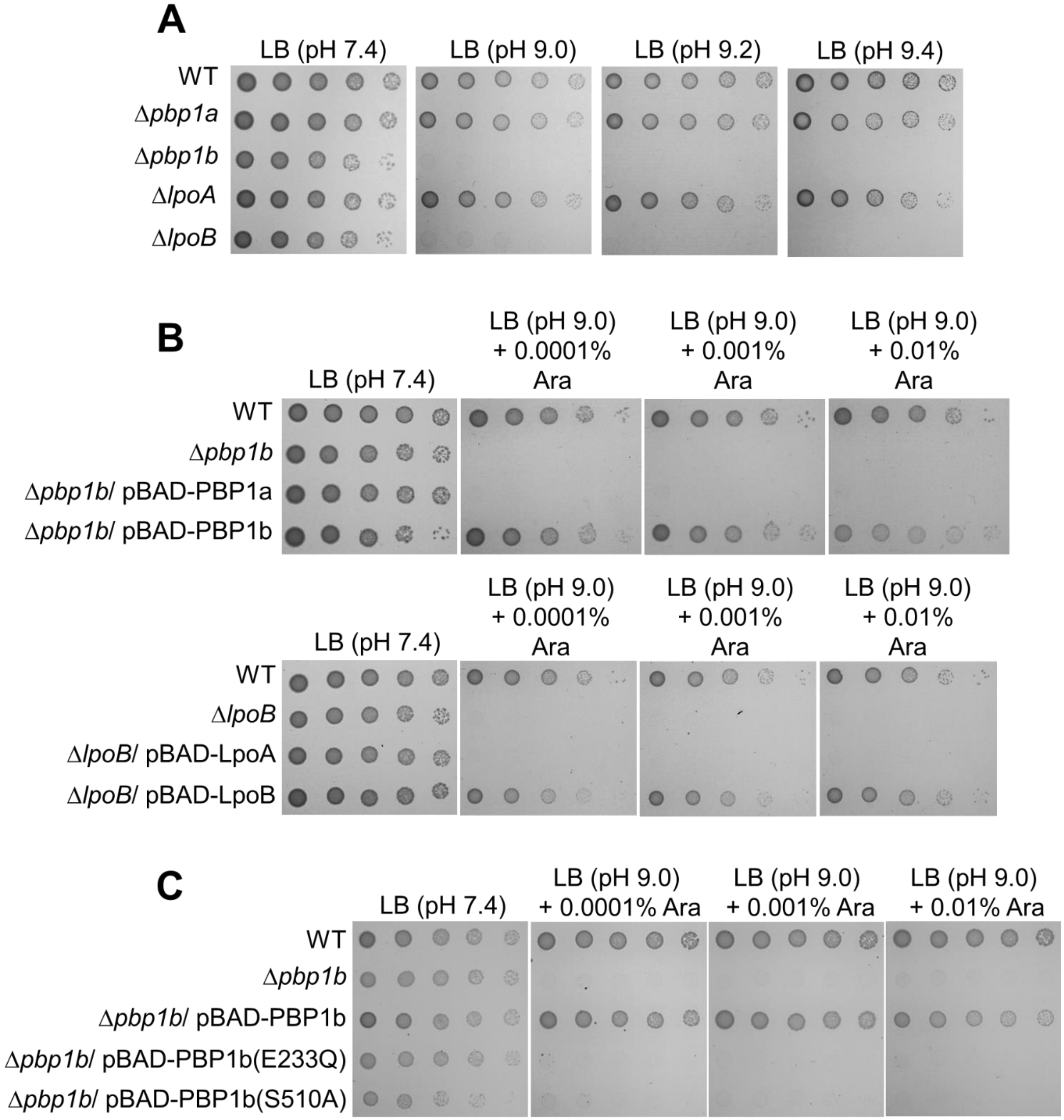

FIG S9 aPBP is required for cell growth under alkaline stress. (A) Sensitivity of *mrcB* and *lpoB* mutants to alkaline stress. The wild-type (WT) and indicated mutant cells were serially diluted from 10^8 to 10^4 cells/ml in 10-fold steps and spotted onto an LB plate or LB plates at the indicated pH. (B) Complementation of alkaline sensitivity of *mrcB* and *lpoB* mutants. The cells of the indicated strains were serially diluted from 10^8 to 10^4 cells/ml in 10-fold steps and spotted onto an LB plate and LB

plates at pH 9.0 containing the indicated concentrations of arabinose. (C) Requirement of the DD-transpeptidase activity of PBP1b for overcoming alkaline stress. The cells of the indicated strains were serially diluted from 10^8 to 10^4 cells/ml in 10-fold steps and spotted onto an LB plate and LB plates at pH 9.0 containing the indicated concentrations of arabinose.

2. Supplementary Tables

Supplementary Table S1. ***Escherichia coli*** **strains and plasmids used in this study.**

Strain or plasmid	Genotype or phenotype	Source or Reference
**Strains**		
MG1655	F⁻ λ⁻ *ilvG*⁻ *rfb*-50 *rph*-1. Wild type *E. coli* K-12	(Blattner *et al.*, 1997)
MG1655 Δ*dacA*	MG1655 *dacA*:: *frt*	(Park *et al.*, 2022)
MG1655 Δ*dacB*	MG1655 *dacB*:: *frt*	(Park *et al.*, 2020)
MG1655 Δ*dacC*	MG1655 *dacC*:: *frt*	(Park *et al.*, 2022)
MG1655 Δ*dacD*	MG1655 *dacD*:: *frt*	(Park *et al.*, 2022)
MG1655 Δ*pbpG*	MG1655 *pbpG*:: *frt*	(Park *et al.*, 2020)
MG1655 Δ*ampH*	MG1655 *ampH*:: *frt*	(Park *et al.*, 2020)
MG1655 Δ*yfeW*	MG1655 *yfeW*:: *frt*	(Park *et al.*, 2022)
MG1655 Δ*dacC* Δ*dacA*	MG1655 *dacC*::*frt dacA*:: *frt*	This study
MG1655 Δ*dacC* Δ*dacA* Δ*dacD*	MG1655 *dacC*::*frt dacA*::*frt dacD*:: *frt*	This study
MG1655 Δ*ldtABCDE*	MG1655 *ldtA*::*frt ldtB*::*frt ldtC*::*frt ldtD*::*frt ldtE*:: *frt*	This study
MG1655 Δ*ldtABCDE* Δ*dacA*	MG1655 *ldtA*::*frt ldtB*::*frt ldtC*::*frt ldtD*::*frt ldtE*::*frt dacA*:: *frt*	This study
MG1655 Δ*ldtABCDE* Δ*dacC*	MG1655 *ldtA*::*frt ldtB*::*frt ldtC*::*frt ldtD*::*frt ldtE*::*frt dacC*:: *frt*	This study
MG1655Δ*mrcA*	MG1655 *mrcA*:: *frt*	(Park *et al.*, 2020)
MG1655Δ*mrcB*	MG1655 *mrcB*:: *frt*	(Park *et al.*, 2020)
MG1655Δ*lpoA*	MG1655 *lpoA*:: *frt*	(Park *et al.*, 2020)

MG1655Δ*lpoB*	MG1655 *lpoB*:: *frt*	(Park *et al.*, 2020)
MG1655 DacA-Flag	MG1655 *dacA*-3xFLAG, Cm^R	(Park *et al.*, 2022)
MG1655 DacA(ΔC)-Flag	MG1655 DacA(1-385)-3xFLAG	(Park *et al.*, 2022)
MG1655 DacC-Flag	MG1655 *dacC*-3xFLAG, Cm^R	This study
MG1655 DacC(ΔC)-Flag	MG1655 DacC(1-378)-3xFLAG, Cm^R	This study
MG1655 DacD-Flag	MG1655 *dacD*-3xFLAG, Cm^R	This study
MG1655 DacA(ΔC)-Flag Δ*dacC*	MG1655 DacA(1-385)-3xFLAG *dacC*:: *frt*	This study
ER2566	F-λ-*fhuA2* [*lon*] *ompT lacZ*::T7 gene 1 gal *sulA11* Δ(*mcrC-mrr*)*114*::*IS10* R(*mcr-73*::*miniTn10*-TetS)2 R(*zgb-210*::*Tn10*)(TetS) *endA1* [*dcm*]	New England Biolabs
**Plasmids**		
pBAD24	Expression vector under control of arabinose-inducible promoter, Amp^r	Addgene
pBAD24(cm)	pBAD24-based expression vector, Cm^r	(Park *et al.*, 2020)
pBAD-Flag	pBAD24(cm)-based expression vector for the 3xFlag tag, Cm^r	(Park *et al.*, 2020)
pBAD-DacA	pBAD24(cm)-based expression vector for DacA, Cm^r	(Park *et al.*, 2022)
pBAD-DacB	pBAD24(cm)-based expression vector for DacB, Cm^r	(Park *et al.*, 2020)
pBAD-DacC	pBAD24(cm)-based expression vector for DacC, Cm^r	(Park *et al.*, 2022)
pBAD-DacD	pBAD24(cm)-based expression vector for DacD, Cm^r	(Park *et al.*, 2022)
pBAD-YfeW	pBAD24(cm)-based expression vector for YfeW, Cm^r	(Park *et al.*, 2022)
pBAD-PbpG	pBAD24(cm)-based expression vector for PbpG, Cm^r	(Park *et al.*, 2020)

pBAD-AmpH	pBAD24(cm)-based expression vector for AmpH, Cm^r	(Park *et al.*, 2020)
pBAD-DacA(ΔC)	pBAD24(cm)-based expression vector for DacA(1-385), Cm^r	This study
pBAD-DacC(ΔC)	pBAD24(cm)-based expression vector for DacC(1-378), Cm^r	This study
pBAD-DacC(S66G)	pBAD24(cm)-based expression vector for DacC(S66G), Cm^r	This study
pBAD-PBP1a	pBAD24(cm)-based expression vector for PBP1a, Cm^r	(Park *et al.*, 2020)
pBAD-PBP1a-His	pBAD24(cm)-based expression vector for PBP1a with N-terminal 6 histidines, Cm^r	This study
pBAD-PBP1b	pBAD24(cm)-based expression vector for PBP1b, Cm^r	(Park *et al.*, 2020)
pBAD-PBP1b-His	pBAD24(cm)-based expression vector for PBP1b with N-terminal 6 histidines, Cm^r	This study
pBAD-PBP2	pBAD24(cm)-based expression vector for PBP2, Cm^r	This study
pBAD-PBP2-His	pBAD24(cm)-based expression vector for PBP2 with N-terminal 6 histidines, Cm^r	This study
pBAD-PBP3	pBAD24(cm)-based expression vector for PBP3, Cm^r	This study
pBAD-PBP3-His	pBAD24(cm)-based expression vector for PBP3 with N-terminal 6 histidines, Cm^r	This study
pBAD-LpoA	pBAD24(cm)-based expression vector for LpoA, Cm^r	(Park *et al.*, 2020)
pBAD-LpoB	pBAD24(cm)-based expression vector for LpoB, Cm^r	(Park *et al.*, 2020)
pBAD-PBP1b(E233Q)	pBAD24(cm)-based expression vector for PBP1b(E233Q), Cm^r	This study
pBAD-PBP1b(S510A)	pBAD24(cm)-based expression vector for PBP1b(S510A), Cm^r	This study

pBAD-Flag-FRT-Kan	pBAD24(cm)-based expression vector containing the 3xFlag tag, FRT-Km^r, Cm^r	(Park *et al.*, 2022)
pKD13	Template plasmid for the amplification of the kanamycin-resistance gene bordered by FRT sites, Km^r	(Datsenko and Wanner, 2000)
pKD46	λ Red recombinase expression plasmid Ts replicon, Amp^r	(Datsenko and Wanner, 2000)
pCP20	FLP helper plasmid Ts replicon, Amp^r, Cm^r	(Datsenko and Wanner, 2000)
pET28a	Expression vector under control of T7 promoter, Km^r	Novagen
pET24a	Expression vector under control of T7 promoter, Km^r	Novagen
pET24a-PBP1b	pET24a-based expression vector for PBP1b, Km^r	(Park *et al.*, 2022)
pET28a-DacA	pET28a-based expression vector for DacA(30-403) with N-terminal 6 histidines, Km^r	(Park *et al.*, 2022)
pET28a-DacA(ΔC)	pET28a-based expression vector for DacA(30-385) with N-terminal 6 histidines, Km^r	(Park *et al.*, 2022)
pET28a-DacC	pET28a-based expression vector for DacC(28-400) with N-terminal 6 histidines, Km^r	This study
pET28a-DacC(ΔC)	pET28a-based expression vector for DacC(28-378) with N-terminal 6 histidines, Km^r	This study
pRE1	Expression vector under control of promoter, Amp^r	(Reddy *et al.*, 1989)
pRE1(kan)	Expression vector under control of promoter, Km^r	(Park *et al.*, 2022)
pRE1-DacA	pRE1-based expression vector for DacA, Km^r	(Park *et al.*, 2022)
pRE1-DacA(ΔC)	pRE1-based expression vector for DacA(1-385), Km^r	(Park *et al.*, 2022)
pRE1-DacA(S73G)	pRE1-based expression vector for DacA(S73G), Km^r	(Park *et al.*, 2022)

pACYC184	A low copy number cloning vector; CmR , TetR	
pACYC184-DacA	pACYC184-based expression vector for DacA, Cm^r	This study
pACYC184-DacA(ΔC)	pACYC184-based expression vector for DacA(1-385), Cm^r	This study

Supplementary Table S2. Oligonucleotides used in this study

Name	Oligonucleotide sequence (5’–3’)	Use(s)
DacC-3xFLAG-F	GGGATTTCGTGATGATGAAATTCCATCAGTGG TTCGGCAGCTGGTTCTCTGTCGACCTGCAGGA TTATAA	Chromosomal 3xFlag tag
DacC-3xFLAG-R	TTAATGACGTATAACCGGATGACGTTTCGCGC CATCCGGTTATCAGAAGATTACGCCCCG CCCTGCCACT	
DacC(ΔC)-3xFLAG-F	CCATTGAGCAGCGTCCGCTGATCGTGATGGA AAATGTGGAAGAGGGCGGAGTCGACCTGCAG GATTATAA	
DacD-3xFLAG-F	GGGAAGGCAGCATGTTTTCTCGCCTGAGTGAT TATTTCCACCATAAGGCCGTCGACCTGCAGG ATTATAA	
DacD-3xFLAG-R	TGATTATGCGAGCAGACTCGCACTCCTGCCA GTCTGCTGCAAAAGAAAGGTTACGCCCCG CCCTGCCACT	
DacC-cfm-F	TGTGTGTGCGTTATTAATCACCAAACTTAT	Chromosomal 3xFlag tag confirm
pET24-DacC-R	GCTCGAATTCGGATCCTGAAGTACGCGCAGA CGCAG	
DacD-3xFLAG-cfm-F	GCAAGAAAATTACTGCGTTGGGGGCAACAA	
DacD-3xFLAG-cfm-R	CTTCCTGCTTAATCTCGTAGTTCATGACGC	
pBAD-DacA-F	CTAGCAGGAGGAATTCATGAATACCATTTTTT CCGC	pBAD24 cloning
pBAD-DacA(ΔC)-R	GCAGGTCGACTCTAGATTAGTTACCTTCCGGG ATTTC	
pBAD-DacC-F	CTAGCAGGAGGAATTCATGACGCAATACTCC TCTC	
pBAD-DacC(ΔC)-R	GCAGGTCGACTCTAGATTATCCGCCCTCTTCC A CATTTT	

pBAD-PBP1a-F	CTAGCAGGAGGAATTCATGAAGTTCGTAAAGTATTT	
pBAD-PBP1a-His-R	GCAGGTCGACTCTAGATCAGTGGTGGTGGTGGTGGTGGTCGTCGTCGTCCTTGAACAATTCCTGTGCCTCGC	
pBAD-PBP1b-F	CTAGCAGGAGGAATTCATGGCCGGGAATGACCGCGA	
pBAD-PBP1b-His-R	GCAGGTCGACTCTAGATCAGTGGTGGTGGTGGTGGTGGTCGTCGTCGTCCTTATTACTACCAAACATATCCT	
pBAD24-PBP2-F	CTAGCAGGAGGAATTCATGAAACTACAGAACTCTTT	
pBAD24-PBP2-R	GCAGGTCGACTCTAGATTAATGGTCCTCCGCTGCGG	
pBAD-PBP2-His-R	GCAGGTCGACTCTAGATCAGTGGTGGTGGTGGTGGTGGTCGTCGTCGTCCTTATGGTCCTCCGCTGCGGCAA	
pBAD-PBP3-F	CTAGCAGGAGGAATTCATGAAAGCAGCGGCGAAAAC	
pBAD-PBP3-R	GCAGGTCGACTCTAGATTACGATCTGCCACCTGTCC	
pBAD-PBP3-His-R	GCAGGTCGACTCTAGATCAGTGGTGGTGGTGGTGGTGGTCGTCGTCGTCCTTCGATCTGCCACCTGTCCCCT	
pET24-PBP1b-F	AAGGAGATATACATATGGCCGGGAATGACCGCGA	
pET-PBP1b-R	GCTCGAATTCGGATCCTGGCAACTCGCCATCCGGTA	
pET28-DacC-F	CGCGCGGCAGCCATATGGCGGAACAAACCGTTGAAG	pET24 and pET28 cloning
pET-DacC-R	GCTCGAATTCGGATCCTGAAGTACGCGCAGACGCAG	

pET-DacC(ΔC)-R	GCTCGAATTCGGATCCTTATCCGCCCTCTTCC ACATTTT	
pACYC184-DacA-F	GCCCATTCGGCCGCAAAGCCGAAGCCAGTA	pACYC184 cloning
pACYC184-DacA-R	GATGCTTGGATCCTGGGGACGGAAATTACA	
pACYC184-DacA(ΔC)-R	GATGCTTGGATCCTTAGTTACCTTCCGGGAT TTC	
DacC(S66G)-F	AAACTGGATCCCGCGGGCCTGACTAAAATC	Point mutant
DacC(S66G)-R	CATGATTTTAGTCAGGCCCGCGGGATCCAG	
PBP1b(E233Q)-F	TACTTTGCTGGCGACACAAGACCGTCATTT	
PBP1b(E233Q)-F	AAATGACGGTCTTGTGTCGCCAGCAAAGTA	
PBP1b(S510A)-F	CGCGTCGTTCGATTGGTGCCCTTGCAAAAC	
PBP1b(S510A)-F	GTTTTGCAAGGGCACCAATCGAACGACGCG	
RT-16S rRNA-F	AAATTGAAGAGTTTGATCATGGCTCAGATT	qRT-PCR
RT-16S rRNA-R	AATGAGCAAAGGTATTAACTTTACTCCCTT	
RT-DacC-F	ATGACGCAAT ACTCCTCTCT CCTTCGTGGT	
RT-DacC-R	CCGGACTG GATAATCACA CCTTTGTTCA AG	

References

Blattner FR, Plunkett G, 3rd, Bloch CA, Perna NT, Burland V, Riley M, Collado-Vides J, Glasner JD, Rode CK, Mayhew GF, Gregor J, Davis NW, Kirkpatrick HA, Goeden MA, Rose DJ, Mau B, Shao Y. 1997. The complete genome sequence of *Escherichia coli* K-12. *Science.* **277**:1453-1462. doi: https://doi.org/

Datsenko KA, Wanner BL. 2000. One-step inactivation of chromosomal genes in *Escherichia coli* K-12 using PCR products. *Proc Natl Acad Sci USA.* **97**:6640-6645. doi: https://doi.org/10.1073/pnas.120163297

Park SH, Kim YJ, Lee HB, Seok YJ, Lee CR. 2020. Genetic Evidence for Distinct Functions of Peptidoglycan Endopeptidases in Escherichia coli. *Front Microbiol.* **11**:565767. doi: https://doi.org/10.3389/fmicb.2020.565767

Park SH, Choi U, Ryu SH, Lee HB, Lee JW, Lee CR. 2022. Divergent Effects of Peptidoglycan Carboxypeptidase DacA on Intrinsic beta-Lactam and Vancomycin Resistance. *Microbiol Spectr.* e0173422. doi: https://doi.org/10.1128/spectrum.01734-22

Reddy P, Peterkofsky A, McKenney K. 1989. Hyperexpression and purification of *Escherichia coli* adenylate cyclase using a vector designed for expression of lethal gene products. *Nucleic Acids Research.* **17**:10473-10488. doi: https://doi.org/10.1093/nar/17.24.10473
